# Incidence of Diabetic Ketoacidosis Among Pediatrics With Type 1 Diabetes Prior to and During COVID-19 Pandemic: A Meta-Analysis of Observational Studies

**DOI:** 10.3389/fendo.2022.856958

**Published:** 2022-03-09

**Authors:** Osamah M. Alfayez, Kholood S. Aldmasi, Nada H. Alruwais, Nouf M. Bin Awad, Majed S. Al Yami, Omar A. Almohammed, Abdulaali R. Almutairi

**Affiliations:** ^1^ Department of Pharmacy Practice, College of Pharmacy, Qassim University, Qassim, Saudi Arabia; ^2^ College of Pharmacy, University of Shaqra, Al Dawadmi, Saudi Arabia; ^3^ Department of Pharmacy Practice and Science, College of Pharmacy, University of Arizona, Tucson, AZ, United States; ^4^ Department of Pharmacy Practice, College of Pharmacy, King Saud bin Abdulaziz University for Health Sciences, Riyadh, Saudi Arabia; ^5^ Department of Clinical Pharmacy, College of Pharmacy, King Saud University, Riyadh, Saudi Arabia; ^6^ Pharmacoeconomics Research Unit, College of Pharmacy, King Saud University, Riyadh, Saudi Arabia; ^7^ Drug Sector, Saudi Food and Drug Authority, Riyadh, Saudi Arabia

**Keywords:** COVID-19, diabetic ketoacidosis, incidence, meta-analysis, pediatrics, systematic review, type 1 diabetes

## Abstract

**Background:**

Previous reports suggest that the Coronavirus Disease-2019 (COVID-19) pandemic might have affected incidences of diabetic ketoacidosis (DKA) and new diagnoses of type 1 diabetes. This systematic review and meta-analysis aimed to estimate the risk of DKA, including severe DKA, during the COVID-19 pandemic versus the prior-to-COVID-19 period among pediatric patients with type 1 diabetes.

**Methods:**

PubMed and EMBASE were searched for observational studies investigating the risk of DKA among pediatric patients with type 1 diabetes during the COVID-19 pandemic and the prior-to-COVID-19 period. A random meta-analysis model was performed to estimate the relative risk of DKA during the COVID-19 pandemic compared to before the pandemic. Subgroup analyses were conducted based on the type 1 diabetes status, established or newly diagnosed. In addition, sensitivity analysis was conducted for studies that reported results from adjusted analysis for potential confounders using fixed effect model.

**Results:**

A total of 20 observational studies reported the risk of DKA, of which 18 reported the risk of severe DKA. The risks of DKA and severe DKA were 35% (RR 1.35, 95%CI 1.2-1.53, *I* ^2^ = 71%) and 76% (RR 1.76, 95%CI 1.33-2.33, *I* ^2 =^ 44%) higher in the during-COVID-19 group compared to the prior-to-COVID-19 group, respectively. Among patients with newly diagnosed type 1 diabetes, the risk of DKA was 44% higher for the during-COVID-19 group compared to the prior-to-COVID-19 group (RR 1.44, 95%CI 1.26-1.65; *I* ^2^ = 64%). Only two studies reported the risk of DKA among patients with established type 1 diabetes and the cumulative risk was not statistically significant. In the sensitivity analysis, four studies reported an adjusted odds ratio (aOR) of the risk of DKA during COVID-19 compared to the prior-to-COVID-19 period. The fixed estimate from the meta-analysis found an increase in the risk of DKA in the during-COVID-19 group compared to the prior-to-COVID-19 group (aOR 2.04, 95%CI 1.66-2.50).

**Conclusions:**

This study showed that DKA risk, especially the risk of severe DKA, has increased significantly during the pandemic. Healthcare systems must be aware and prepared for such an increase in DKA cases and take all necessary measures to prevent future spikes during the pandemic.

**Systematic Review Registration:**

https://www.crd.york.ac.uk/prospero/display_record.php?RecordID=272775, identifier PROSPERO [CRD42021272775].

## Introduction

Diabetic ketoacidosis (DKA) is a life-threatening complication of diabetes that can occur at type 1 diabetes onset (1, 2). The International Society of Pediatric and Adolescent Diabetes defined DKA patients as having a blood glucose level greater than 200 mg/dl, a pH level under 7.3, and a bicarbonate level under 15 mmol/L. Severe DKA, however, is recognized by a decline in pH level to under 7.1 or potentially a bicarbonate level under 5 mmol/L (3). The incidence rate of DKA at type 1 diabetes onset ranged from 13 to 80%, requiring hospitalization in most cases and leading to the consumption of more healthcare resources (3–5). The recently reported prevalence of DKA among children with newly diagnosed type 1 diabetes was around 30%, with much variation in the prevalence between 13 countries in the study (6).

Patients with diabetes are at greater risk of infection relative to the general population, and the risk is higher for patients with type 1 diabetes compared to patients with type 2 diabetes (7). The Coronavirus Disease-2019 (COVID-19) pandemic has dramatically affected the lifestyle of patients and their access to healthcare services worldwide, including delayed diagnosis or management of chronic diseases, such as type 1 diabetes (8, 9). Moreover, several studies reported an increase in DKA and severe DKA cases among the pediatric population (10–17). Also, some studies reported a possible increase in type 1 diabetes cases during the pandemic (11, 12, 18). Therefore, we conducted a comprehensive systematic review and meta-analysis to estimate the risk of DKA, including severe DKA, among patients with type 1 diabetes prior to and during the COVID-19 pandemic.

## Methods

### Search Strategy and Databases

A systematic literature search was conducted by KSA, NHA, and NMB for observational studies using the PICO (population, intervention, comparison, outcome) framework (P: pediatric patients with type 1 diabetes, I: during the COVID-19 pandemic, C: prior to COVID-19, O: incidences of DKA). The electronic databases *PubMed* and *EMBASE* were searched from inception to December 28, 2021. The *Elsevier Coronavirus Research Repository Hub* was also searched for potentially eligible studies. The search strategy and keywords are available in **Supplemental Table 1**. This systematic review and meta-analysis was registered with PROSPERO (CRD42021272775), and the manuscript was prepared based on the Meta-Analysis of Observational Studies in Epidemiology (MOOSE) guideline (19).

### Study Selection, Data Extraction, and Quality Assessment

All retrieved citations, after removing duplicates from the Rayyan software (20), were independently screened by the three investigators for eligibility; initially through titles and abstracts, then through a full-text review. Studies published as an abstract or in non-English language were excluded. Disagreements were resolved by consensus. Two investigators (KSA and NHA) extracted the following data into an Excel sheet: primary author’s last name, year of publication, location of the study, study period, key inclusion/exclusion criteria, number of patients with type 1 diabetes, study period, and number of patients with DKA and severe DKA. Two other investigators (ARA and OMA) checked the extracted data and independently performed the quality assessment of included articles using the Newcastle-Ottawa Scale (NOS) (21, 22). Disagreements were resolved by consensus.

### Data Synthesis and Analysis

The primary outcome of the analysis estimated the risk of DKA among pediatric patients with type 1 diabetes in the during-COVID-19 pandemic group relative to the prior-to-COVID-19 group using the risk ratio (RR) with a 95% confidence interval (95% CI). The secondary outcome is the relative risk of severe DKA in the during-COVID-19 group versus the prior-to-COVID-19 group. We performed the meta-analysis with a random effect model using R version 4.0.4 and presented the results on a forest plot, including the heterogeneity I² statistics. Egger’s test was employed to assess the potential for publication bias. We performed subgroup analyses based on type 1 diabetes status (newly or established diagnoses) and sensitivity analyses for studies that reported adjusted point estimates (RR or OR) using the fixed effect model.

## Results

### Characteristics of Included Studies

**Figure 1:**
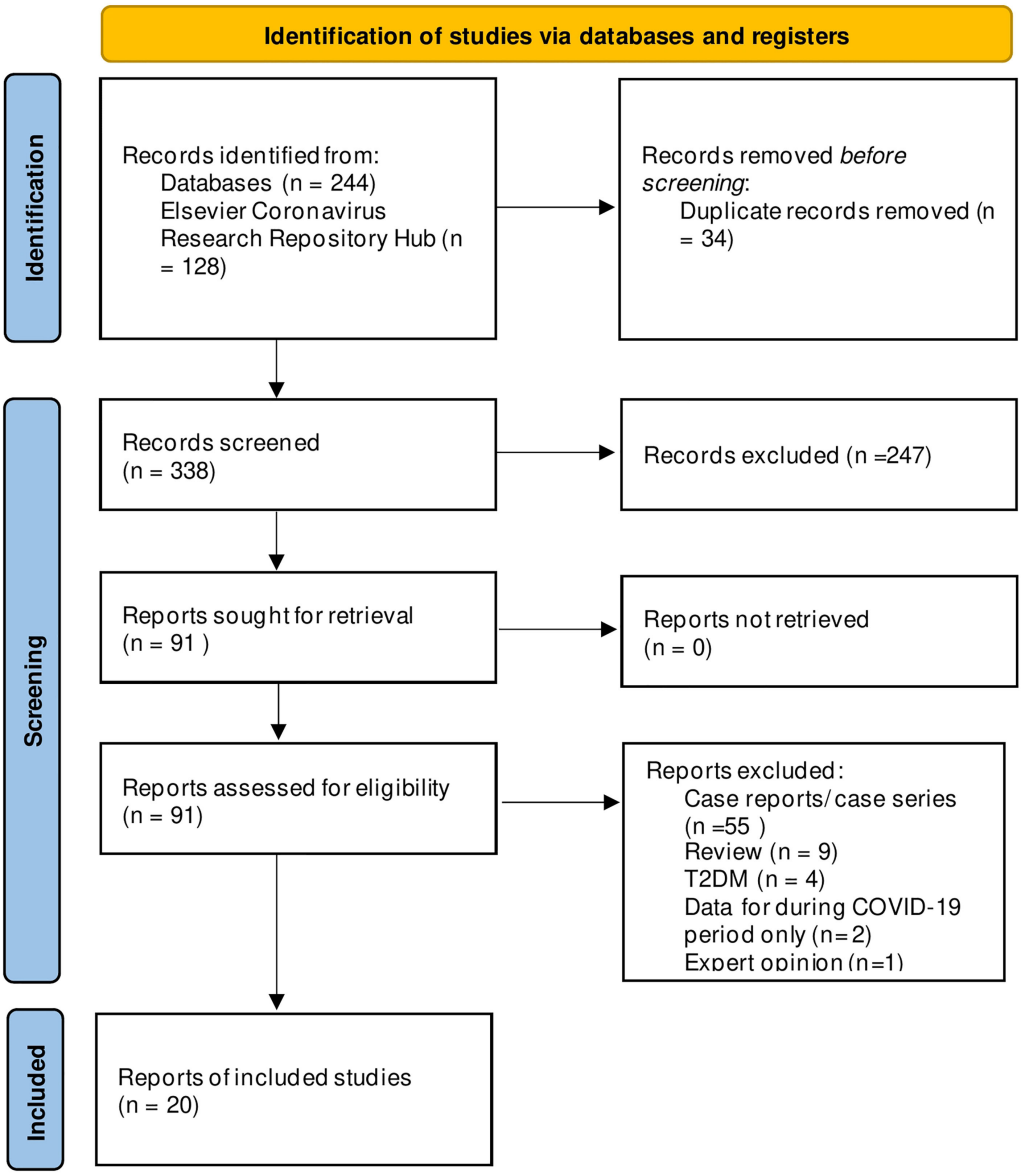
PRISMA Flowchart of included and excluded studies. Copyright statement: this PRISMA diagram contains public sector information licensed under the Open Government Licence v3.0. Adapted From: Moher et al. (23).

The systematic search yielded 372 citations, and 20 studies met the inclusion criteria (**Figure 1**) (10–18, 24–34). These studies included 37,174 patients with type 1 diabetes in the prior-to-COVID-19 pandemic group and 27,812 patients in the during-COVID-19 group. Most of the studies were conducted in European countries from hospitals or tertiary care centers (**Table 1**).

**Table 1:** Characteristics of the included studies.

Study	Location	COVID-19		Patient included	Setting
		During (N)	Prior (N)		
Dżygało, 2020 (17)	Poland	34	52	≤18 years	Single center
Kamrath, 2020 (10)	Germany	532	954	≤18 years	Multicenter
Rabbone, 2020 (18)	Italy	160	208	<15 years	Multicenter
Alaqeel, 2021 (32)	Saudi Arabia	106	154	1–14 years	Multicenter
Boboc, 2021 (11)	Romania	147	312	<18 years	Single center
Bogale, 2021 (24)	USA	42	370	≤18 years	Single center
Danne, 2021 (25)	Worldwide	25543	31258	≤21 years	Multicenter
Dilek, 2021 (12)	Turkey	74	46	<18 years	Single center
Hawkes, 2021 (26)	USA, Europe	73	92	<18 years	Single center
Ho, 2021 (13)	Canada	107	114	<18 years	Multicenter
Jacob, 2021 (14)	Israel	150	154	≤18 years	Multicenter
Lawrence, 2021 (15)	Australia	11	42	<18 years	Single center
McGlacken, 2021 (16)	UK	17	30	<18 years	Multicenter
Salmi, 2021 (27)	Finland	84	231	≤15 years	Multicenter
Zubkiewicz-Kucharska, 2021 (28)	Poland	30	1961	<18 years	Multicenter
Al‐Abdulrazzaq, 2021 (29)	Kuwait	324	303	≤12 years	Multicenter
Goldman, 2021 (30)	Israel	146	364	<18 years	Multicenter
Kostopoulou, 2021 (31)	Greece	21	17	<18 years	Multicenter
Mameli, 2021 (32)	Italy	201	502	<18 years	Multicenter
Mi Seon Lee, 2021 (33)	Korea	10	10	<19 years	Single center

### Risk of Bias Assessment

All the included studies had more than 7 points on the NOS scale, suggesting good (7–9) quality (**Supplemental Table 2**). However, only seven studies demonstrated a good quality on the comparability domain due to the adjustment for the baseline characteristics between the two groups (during-COVID-19 and prior-to-COVID-19).

### Risk of DKA

**Figure 2:**
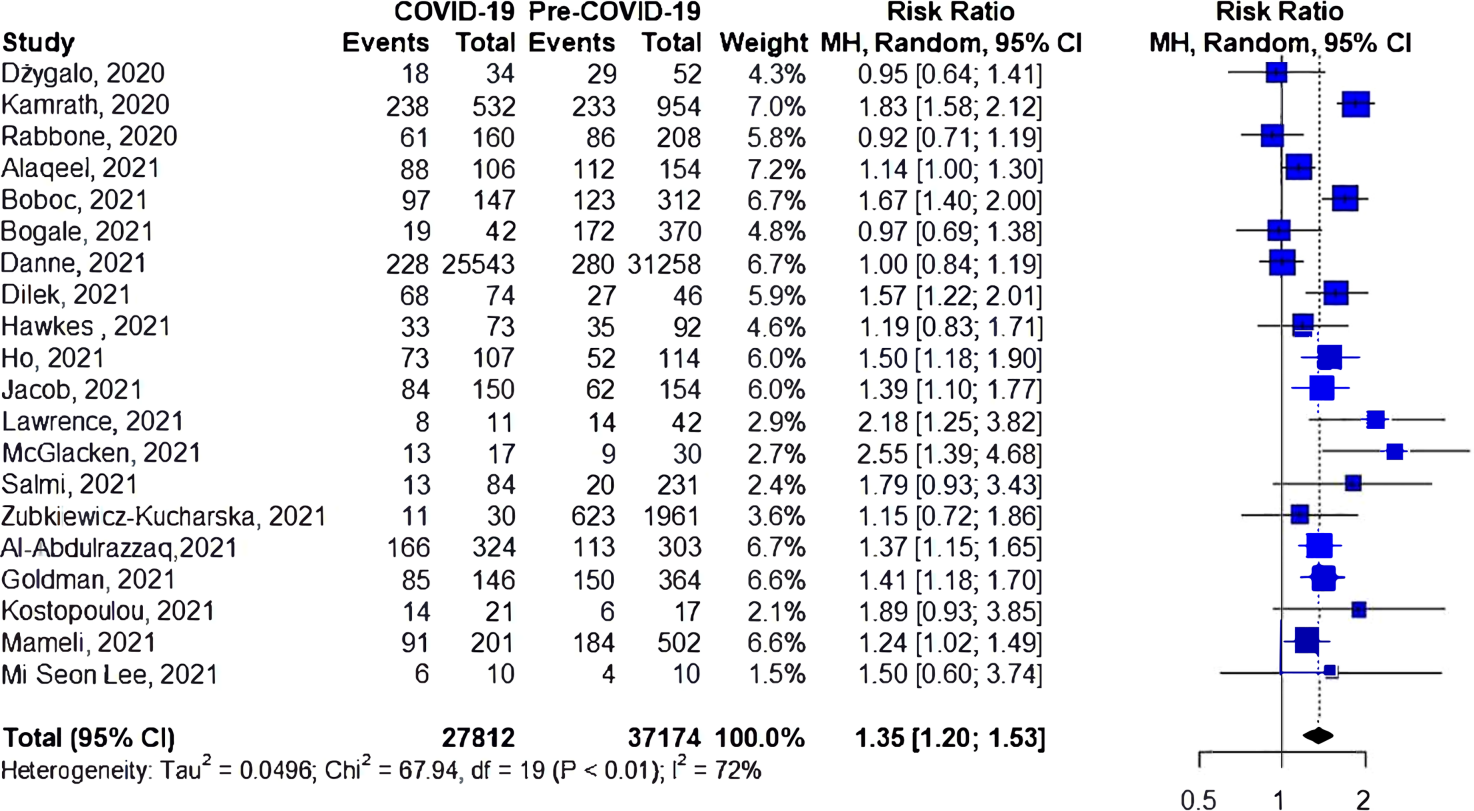
Risk of DKA among patients with type 1 diabetes.

A total of 20 studies investigated the risk of DKA during COVID-19 compared to the prior-to-COVID-19 period (**Figure 2**). Seven studies have shown an increase in the risk of DKA during the pandemic. The cumulative risk of DKA was 35% higher for the during-COVID-19 group compared to the prior-to-COVID-19 group (RR 1.35, 95%CI 1.20-1.53) but with significant heterogeneity (I^2^ = 71%, *p*<0.01).

### Risk of Sever DKA

**Figure 3:**
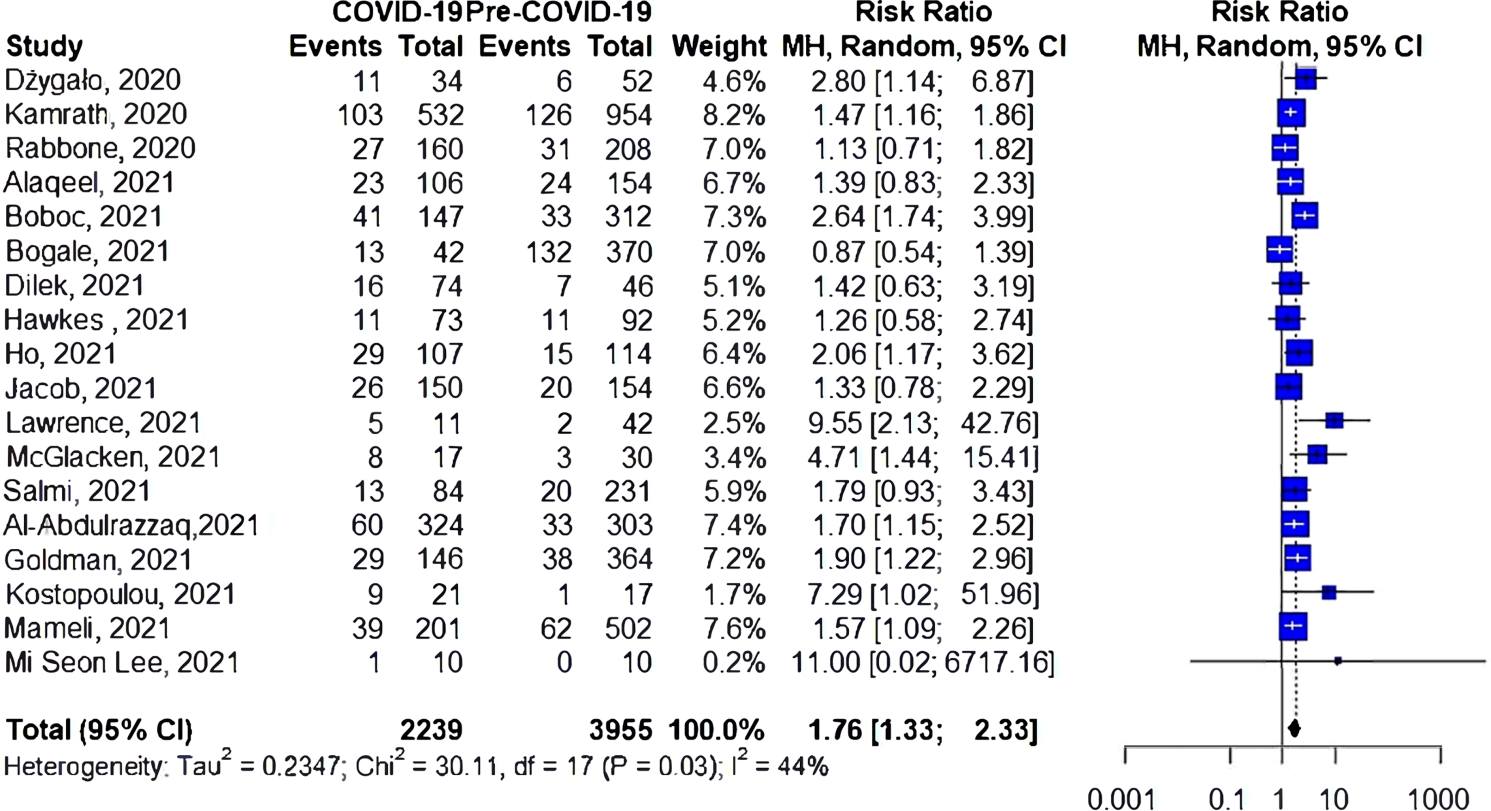
Risk of severe DKA among patients with type 1 diabetes.

Eighteen studies have investigated the risk of severe DKA during COVID-19 compared to the prior-to-COVID-19 period (**Figure 3**). Ten studies have shown an increase in the risk of severe DKA during the pandemic. The cumulative risk of severe DKA was 76% higher for the during-COVID-19 group compared to the prior-to-COVID-19 group (RR 1.76, 95%CI 1.33-2.33, I^2 ^= 44%, *p=0.03*).

### Subgroup Analysis

#### Risk of DKA Among Patients With Newly Diagnosed Type 1 Diabetes

**Figure 4:**
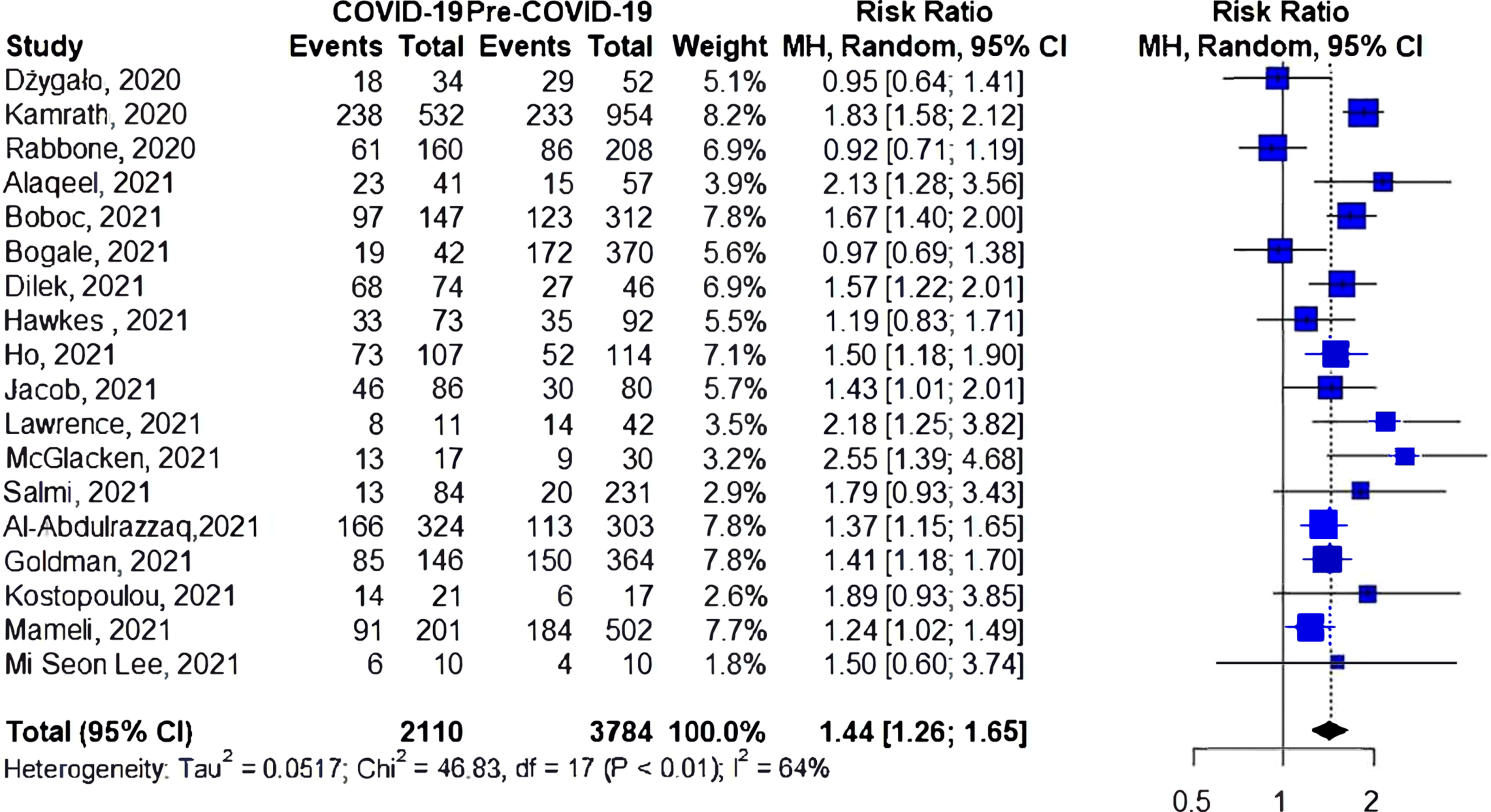
Risk of DKA among patients with newly diagnosed type 1 diabetes.

A total of 18 studies investigated the risk of DKA among patients with newly diagnosed type 1 diabetes during COVID-19 compared to the prior-to-COVID-19 period (**Figure 4**), with eleven reporting an increase in the risk of DKA during the pandemic. The cumulative risk showed a 44% increase in the risk of DKA for the during-COVID-19 compared to the prior-to-COVID-19 groups (RR 1.44, 95%CI 1.26-1.65), again with significant heterogeneity (I^2 ^= 64%, *p*<0.01).

#### Risk of DKA Among Patients With Established Type 1 Diabetes

**Figure 5:**
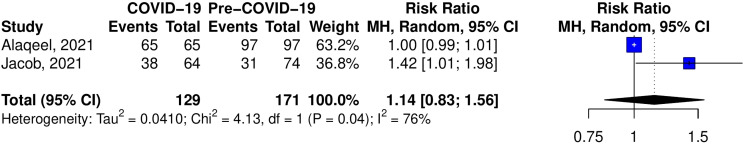
Risk of DKA among patients with established type 1 diabetes.

Two studies have reported the risk of DKA among patients with established type 1 diabetes (**Figure 5**); one of them found an increase in the risk of DKA during the pandemic. However, the cumulative risk of DKA was insignificant (RR 1.14, 95%CI 0.83-1.56, I^2 ^= 76%, *p*=0.04).

### Sensitivity Analysis

Four studies reported an adjusted odds ratio (aOR) of the risk of DKA during COVID-19 compared to the prior-to-COVID-19 period (**Supplemental Figure 1**). The fixed estimate from the meta-analysis showed an increase in the risk of DKA for the during-COVID-19 group compared to the prior-to-COVID-19 group (aOR 2.04, 95%CI 1.66-2.50). Conversely, three studies reported an adjusted relative risk (aRR) of DKA during COVID-19 compared to the prior-to-COVID-19 period (**Supplemental Figure 2**). The fixed estimate from the meta-analysis showed an increase in the risk of DKA for the during-COVID-19 group compared to the prior-to-COVID-19 group (aRR 1.27, 95%CI 1.18-1.36).

### Publication Bias

The publication bias test was performed for all outcomes except for the subgroup analysis of the established type 1 diabetes cohort, which only included two studies. The results of Eager’s test did not show potential for publication bias.

## Discussion

This meta-analysis aimed to estimate the risk of DKA, including severe DKA, among pediatric patients with type 1 diabetes during COVID-19 pandemic compared to prior-to-COVID-19. The cumulative risks of DKA and severe DKA were 35% and 76% higher, respectively, for the during-COVID-19 period compared to prior to COVID-19. The cumulative risk of DKA among patients with newly diagnosed type 1 diabetes showed a 44% increased risk of DKA for the during-COVID-19 compared to the prior-to-COVID-19 groups.

Several hypotheses were raised to explain the increase in incidences of DKA and severe DKA during the pandemic. A delay in seeking medical attention was suggested by several studies (10, 35, 36). Such a delay was attributed to fear of infection, cancellation of several medical services, or the closing of some centers due to an increase in infections among medical staff and admitted patients (10, 35, 37–39). For those newly diagnosed with type 1 diabetes presenting with DKA, it is hypothesized that delay in seeking medical help will be preceded by a longer duration of type 1 diabetes symptoms. However, several studies have reported that the duration of symptoms during the pandemic were comparable to duration prior to COVID-19 (11, 17, 28, 40). This finding suggests that the delay in diagnosing type 1 diabetes is not the only cause for an increased risk of DKA. This result might also be supported by the reported no-change or unexpected lower HbA1c levels during the pandemic (25, 28). That said, a limitation does exist regarding the duration of symptoms given that the information provided in the studies depends on parents’ ability to recall those symptoms.

Most of the studies included in this meta-analysis investigated those who were newly diagnosed with type 1 diabetes, which makes it unlikely that worsening glycemic control during the pandemic led to the increase in DKA cases. Only two studies have reported DKA cases among patients with a history of type 1 diabetes. Jacob et al. reported a statistically significant increase in DKA during the pandemic (14), while Alaqeel et al. showed no difference in DKA cases in those with preexisting type 1 diabetes (34). Although some studies have reported a decrease in physical activities during the lockdown period (25, 41), many studies have observed good glycemic control during the lockdown period, which might be a result of close parental supervision during the lockdown or the increased use of diabetes-related technology in some countries (25, 42–45).

Another possible factor could be an increase in the incidence of type 1 diabetes among children during the pandemic. However, most of the studies investigating this idea did not report a significant increase in the incidence of type 1 diabetes during the pandemic. Boboc et al. reported a 30% overall increase in incidences of type 1 diabetes between March 2020 and February 2021 (11). Interestingly, incidences were found to be lower during the early weeks of the pandemic (March to April 2020). Rabbone et al. also reported a decrease in new cases of type 1 diabetes during the pandemic from February 2020 to April 2020 (18). Such an early decrease in incidences of type 1 diabetes during the pandemic might be attributed to lower overall exposure to viral infections during the lockdown period, as viral infections are known risk factor for developing type 1 diabetes (46–48). Moreover, it is also reported that new type 1 diabetes cases are usually higher in the winter season compared to summer (49–51). Thus, it is unlikely that the seasonality of type 1 diabetes is the reason for the decrease in type 1 diabetes incidences reported by those studies (11, 18). Overall, the results regarding the changes in the incidence of type 1 diabetes during the pandemic are inconclusive, making larger studies with a longer duration warranted.

COVID-19 infection has resulted in several complications. Some reports suggest that the virus might be able to affect the pancreas, resulting in a dysregulation of glucose metabolism (52, 53). A UK study suggested a possible link between COVID-19 infection and new-onset of type 1 diabetes or severe DKA (40). However, this study was limited by its small sample size. Conversely, a German study showed that COVID-19 infection did not increase type 1 diabetes cases when there was no evidence of autoimmunity. Importantly, this study only covered the first wave of the pandemic in Germany (approximately four months period) (54). Thus, possible links cannot be entirely ruled out, but larger studies are needed to confirm them. Unfortunately, among the studies included in this meta-analysis, only a small number of patients had COVID-19, and data regarding prior exposure to the virus are limited, making it difficult to attribute such an increase in DKA cases to current or prior COVID-19 infection.

Regardless of the actual cause of increased DKA during the pandemic, DKA remains to be a serious life-threatening complication that must be prevented. Early detection of type 1 diabetes symptoms and early diagnoses is key in preventing DKA. Overall, incidences of DKA at type 1 diabetes diagnosis is a challenge (2, 6, 55, 56), despite the pandemic. Missed diagnoses of type 1 diabetes by healthcare providers have been documented (56, 57). Children diagnosed with type 1 diabetes were more likely to have visited their primary care providers within the 30 days prior to diagnosis (57), which indicates missed opportunities for early detection of type 1 diabetes symptoms.

Efforts should be directed toward increasing awareness of healthcare providers, patients, and families regarding the symptoms of type 1 diabetes. Prevention campaigns have proven useful in reducing the prevalence rate of DKA in newly diagnosed children with type 1 diabetes (58–60). Cherubini et al. study suggested several actions that can optimize awareness campaigns such as targeting families of children under the age of 15 years, smaller geographic areas and family pediatricians in addition to utilizing innovative communication tools (61). Although public health campaigns might be challenging during pandemics given the public is receiving large amount of health information, they could be useful as a public health tool during such pandemic periods when DKA is on the rise.

In the past few years there have been significant improvements in identifying those with high risk for developing type 1 diabetes. Moreover, screening for type 1 diabetes through islet autoantibodies and genetic testing is currently reserved for research (62). However, one main advantage of such screening at the population level would be the reduction of DKA at type 1 diabetes onset (63). This was observed in two studies were significant reductions in DKA frequency was noted (64, 65). However, the cost-effectiveness of screening needs to be assessed. Given the significant increase in DKA and severe DKA cases observed during the COVID-19 pandemic, screening for type 1 diabetes during or right after such pandemics might be considered as a potential tool to aid preventing DKA cases in the future.

This study is not without limitations. All the included studies are observational and had some degree of heterogeneity. Seven studies demonstrated a good quality in the comparability domain. Differences in inclusion criteria, analyzed periods, and number of subjects included might have played a role in the observed heterogeneity. Few studies presented an adjustment for potential confounders and included them in the sensitivity analysis. Only two studies reported DKA cases for those with a history of type 1 diabetes, which made it impossible to assess if poor glycemic control might have played a role in increased DKA cases. Besides that, the mean duration of type 1 diabetes was only reported in one study. Insufficient data were reported regarding COVID-19 infection status among the included studies which made it impractical to perform a subgroup analysis for those with positive COVID-19 infection at type 1 diabetes diagnosis.

In conclusion, the results of this meta-analysis showed a statistically significant increase in DKA and severe DKA risk among pediatrics during the pandemic in comparison to prior to the pandemic period. Such an increase might be attributed to several factors that might differ in magnitude from one country to another. Healthcare systems must be aware and prepared for such an increase in the risk of DKA or severe DKA during similar pandemic conditions. Timely access to healthcare, an increase in public and healthcare providers’ awareness of type 1 diabetes symptoms through public health educational and screening campaigns, and proper diabetes management during pandemics or similar situations remain important and key to avoiding similar spikes in incidences of DKA or severe DKA in the future.

## Data Availability Statement

The original contributions presented in the study are included in the article/**Supplementary Material**. Further inquiries can be directed to the corresponding author.

## Author Contributions

OMA and ARA contributed to the conception and design of this study. KSA, NHA, and NMB conducted the literature search. KSA and NaA performed the data extraction. ARA did the statistical analysis and wrote the methods section with inputs from MA, OAA, and OMA. All authors contributed to writing the manuscript. All authors revised and approved the final version of the manuscript. OMA is the guarantor of this work.

## Funding

This work was supported by the Researcher Supporting Project number (RSP-2021/77), King Saud University, Riyadh, Saudi Arabia. The funding agency had no role in designing the study, conducting the analysis, interpreting the data or writing the manuscript.

## Author Disclaimer

The contents of this manuscript are solely the authors’ views and may not be understood or quoted as being made on behalf of or reflecting the position of the Saudi Food and Drug Authority.

## Conflict of Interest

The authors declare that the research was conducted in the absence of any commercial or financial relationships that could be construed as a potential conflict of interest.

## Publisher’s Note

All claims expressed in this article are solely those of the authors and do not necessarily represent those of their affiliated organizations, or those of the publisher, the editors and the reviewers. Any product that may be evaluated in this article, or claim that may be made by its manufacturer, is not guaranteed or endorsed by the publisher.
