# Supplementary material for: Incidence of Diabetic Ketoacidosis Among Pediatrics With Type 1 Diabetes Prior to and During COVID-19 Pandemic: A Meta-Analysis of Observational Studies
Source: Front Endocrinol (Lausanne). 2022 Mar 9;13:856958. doi: 10.3389/fendo.2022.856958 (PMC8959619; doi:10.3389/fendo.2022.856958)
Supplement: Supplementary file 1 [file DataSheet_1.docx]

**Supplemental Materials**

**Table S1: Search strategy**

| **1-PubMed**  (((acidoses, diabetic[MeSH Terms] OR acidosis, diabetic[MeSH Terms] OR diabetic ketoacidoses[MeSH Terms] OR diabetic ketoacidosis[MeSH Terms] OR diabetic ketoacid*) AND (type 1 diabetes mellitus[MeSH Terms] OR diabetes mellitus, type 1[MeSH Terms] OR diabetes mellitus, type i[MeSH Terms] OR brittle diabetes mellitus[MeSH Terms] OR diabetes mellitus, brittle[MeSH Terms] OR "juvenile onset diabetes" OR "brittle diabetes" OR "insulin dependent diabetes" OR iddm OR "autoimmun* diabet*" OR "sudden onset diabetes" OR T1DM OR T1D OR "insulin* depend*" OR "insulin?depend*" OR "typ? 1 diabet*" OR "typ?1 diabet*" OR "auto-immun* diabet*") AND (coronavirus[MeSH Terms] OR coronavirus, sars[MeSH Terms] OR ncov[MeSH Terms] OR “COVID-19” OR “COVID 19” OR "COVID19" OR “COVID-2019” OR "COVID 2019"OR "COVID2019" OR “COVID” OR "corona virus*" OR "coronavirus*" OR "coronavirus 19" OR "coronavirus 2019" OR "corona virus 19" OR "corona virus 2019" OR "2019 nCOV" OR "2019nCov" OR "nCov 2019" OR "2019-ncov" OR "ncov19" OR "ncov-19" OR "2019-novel cov" OR "SARS-Cov2" OR "SARS-Cov 2" OR "SARSCoV-2" OR "SARSCoV2" OR "SARSCoV-2") |
| --- |
| **2- EMBASE**  'diabetes mellitus, type 1'/exp OR 'type 1 diabetes mellitus':ab,ti OR 'juvenile onset diabetes':ab,ti OR 'brittle diabetes':ab,ti OR 'insulin dependent diabetes':ab,ti OR iddm:ab,ti OR 'autoimmune diabetes':ab,ti OR 'sudden onset diabetes':ab,ti AND 'diabetic ketoacidosis'/exp OR 'diabetic acidosis'/exp OR 'diabetic ketosis'/exp AND 'coronaviridae'/de OR 'coronavirinae'/de OR 'coronaviridae infection'/de OR 'coronavirus disease 2019'/exp OR 'coronavirus infection'/de OR 'SARS‐related coronavirus'/de OR 'Severe acute respiratory syndrome coronavirus 2'/exp OR '2019 nCoV':ti,ab,kw OR 2019nCoV:ti,ab,kw OR coronovir*:ti,ab,kw OR COVID:ti,ab,kw OR COVID19:ti,ab,kw OR HCoV*:ti,ab,kw OR 'nCov 2019':ti,ab,kw OR 'SARS CoV2':ti,ab,kw OR 'SARS CoV 2':ti,ab,kw OR SARSCoV2:ti,ab,kw OR 'SARSCoV 2':ti,ab,kw |

**Table S2: Risk of bias assessment**

| Study | Selection | Comparability | Exposure | note |
| --- | --- | --- | --- | --- |
| Dżygało, 2020 | ★★★★ |  | ★★★ | No adjustment |
| Kamrath, 2020 | ★★★★ | ★ | ★★★ | adjusting for age, sex, and immigrant background |
| Rabbone, 2020 | ★★★★ |  | ★★★ | No adjustment |
| Alaqeel, 2021 | ★★★★ | ★ | ★★★ | adjusting for age, and sex, |
| Boboc, 2021 | ★★★★ | ★★ | ★★★ | Propensity score for all characteristic variables |
| Bogale, 2021 | ★★★★ | ★★ | ★★★ | Multivariable (age, sex, BMI percentile, insurance coverage, primary care provide, A1C, altered mental status, Autism) |
| Danne, 2021 | ★★★★ | ★ | ★★★ | Adjusted for gender, age-and diabetes duration-groups |
| Dilek, 2021 | ★★★★ |  | ★★★ | No adjustment |
| Hawkes , 2021 | ★★★★ |  | ★★★ | No adjustment |
| Ho, 2021 | ★★★★ |  | ★★★ | No adjustment |
| Jacob, 2021 | ★★★★ |  | ★★★ | No adjustment |
| Lawrence, 2021 | ★★★★ |  | ★★★ | No adjustment |
| McGlacken, 2021 | ★★★★ |  | ★★★ | No adjustment |
| Salmi, 2021 | ★★★★ |  | ★★★ | No adjustment |
| Zubkiewicz-Kucharska, 2021 | ★★★★ |  | ★★★ | No adjustment |
| Al‐Abdulrazzaq, 2021 | ★★★★ | ★ | ★★★ | Adjusting for age, gender, BMI z‐score and family history of  diabetes |
| Goldman, 2021 | ★★★★ | ★★ | ★★★ | Adjusting for age at diabetes onset, sex, and socioeconomic position (SEP) index. |
| Kostopoulou, 2021 | ★★★★ |  | ★★★ | No adjustment |
| Mameli, 2021 | ★★★★ |  | ★★★ | No adjustment |
| Mi Seon Lee, 2021 | ★★★★ |  | ★★★ | Age-matched |

**
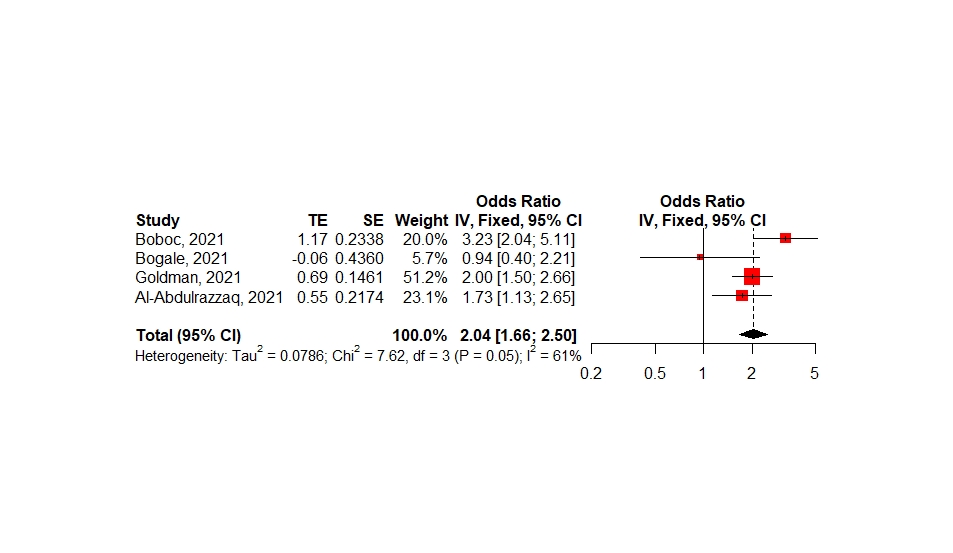
**

**Figure S1:** Forest plots of the risk of DKA in studies that reported adjusted OR

**
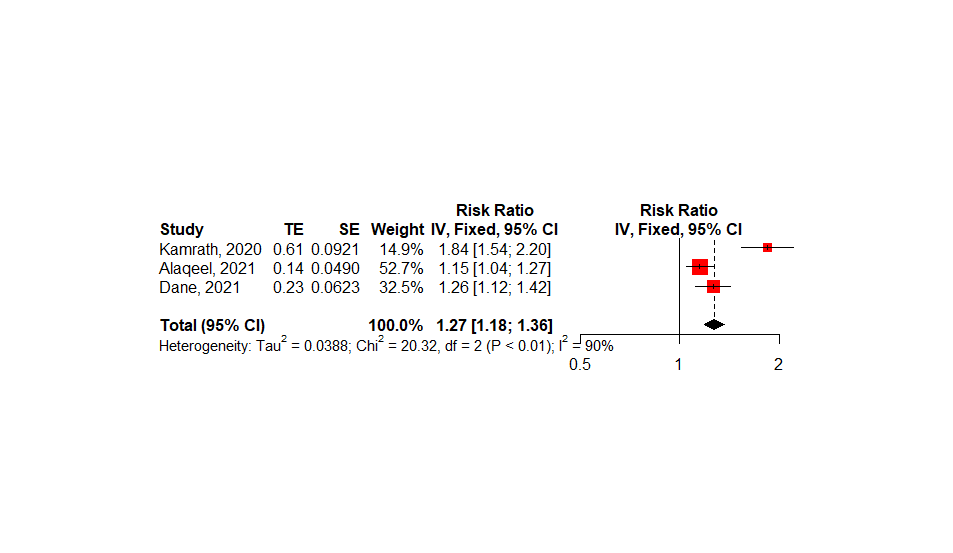
**

**Figure S2:** Forest plots of the risk of DKA in studies that reported adjusted RR
