# Supplementary material for: Incidence of Diabetic Ketoacidosis Among Pediatrics With Type 1 Diabetes Prior to and During COVID-19 Pandemic: A Meta-Analysis of Observational Studies
Source: Front Endocrinol (Lausanne). 2022 Mar 9;13:856958. doi: 10.3389/fendo.2022.856958 (PMC8959619; doi:10.3389/fendo.2022.856958)
Supplement: Supplementary file 2 [file DataSheet_2.docx]

**MOOSE Checklist**

From: Stroup DF, Berlin JA, Morton SC, et al (2000) Meta-analysis of observational studies in epidemiology: A proposal for reporting. JAMA 283:2008–2012. doi:10.1001/jama.283.15.2008.

|  | Reported on page | Comments |
| --- | --- | --- |
| **Reporting of background should include** | | |
| Problem definition | P3, L79-85 |  |
| Hypothesis statement | P3, L79-85 |  |
| Description of study outcome(s) | P3, L73-78 |  |
| Type of exposure or intervention used | P3, L86-87 |  |
| Type of study designs used | P3, L86 |  |
| Study population | P3, L87 |  |
| **Reporting of search strategy should include** | | |
| Qualifications of searchers (e.g. librarians and investigators) | P3, L91 |  |
| Search strategy, including time period used in the synthesis and key words | P3, L94 |  |
| Effort to include all available studies, including contact with authors | P3, L95 |  |
| Databases and registries searched | P3, L93-94 |  |
| Search software used, name and version, including special features used (e.g. explosion) | P4, L102 |  |
| Use of hand searching (e.g. reference lists of obtained articles) | P3, L96-97 |  |
| List of citations located and those excluded, including justification | Figure 1 |  |
| Method of addressing articles published in languages other than English | P3, L96 |  |
| Method of handling abstracts and unpublished studies | P3, L96 |  |
| Description of any contact with authors | NA |  |
| **Reporting of methods should include** | | |
| Description of relevance or appropriateness of studies assembled for assessing the hypothesis to be tested | P4, L101-110 |  |
| Rationale for the selection and coding of data (e.g. sound clinical principles or convenience) | P4, L101-110 |  |
| Documentation of how data were classified and coded (e.g. multiple raters, blinding and interrater reliability) | P4, L101-110 |  |
| Assessment of confounding (e.g. comparability of cases and controls in studies where appropriate) | P4, L101-110 |  |
| Assessment of study quality, including blinding of quality assessors, stratification or regression on possible predictors of study results | P4, L110 |  |
| Assessment of heterogeneity | P4, L117 |  |
| Description of statistical methods (e.g. complete description of fixed or random effects models, justification of whether the chosen models account for predictors of study results, dose-response models, or cumulative meta-analysis) in sufficient detail to be replicated | P4, L111-120 |  |
| Provision of appropriate tables and graphics | Figure2-5 |  |
| **Reporting of results should include** | | |
| Graphic summarizing individual study estimates and overall estimate | Figure2-5 |  |
| Table giving descriptive information for each study included | Table 1 |  |
| Results of sensitivity testing (e.g. subgroup analysis) | P6, L155-163 |  |
| Indication of statistical uncertainty of findings | P5-6 |  |
| **Reporting of discussion should include** | | |
| Quantitative assessment of bias (e.g. publication bias) | P6, L164-167 |  |
| Justification for exclusion (e.g. exclusion of non-English language citations) | NA |  |
| Assessment of quality of included studies | P6, L127-131 |  |
| **Reporting of conclusions should include** | | |
| Consideration of alternative explanations for observed results | P9-10 |  |
| Generalization of the conclusions (i.e. appropriate for the data presented and within the domain of the literature review) | P9-10 |  |
| Guidelines for future research | P9-10 |  |
| Disclosure of funding source | P10 |  |
